# Unpicking the signal thread of the sector web spider *Zygiella x-notata*

**DOI:** 10.1098/rsif.2015.0633

**Published:** 2015-12-06

**Authors:** Beth Mortimer, Chris Holland, James F. C. Windmill, Fritz Vollrath

**Affiliations:** 1 Department of Zoology, University of Oxford, Oxford OX1 3PS, UK; 2 Department of Materials Science and Engineering, University of Sheffield, Sheffield S1 3JD, UK; 3 Department of Electronic and Electrical Engineering, University of Strathclyde, Glasgow G1 1XW, UK

**Keywords:** vibration, spider, remote sensing, silk

## Abstract

Remote sensing allows an animal to extend its morphology with appropriate conductive materials and sensors providing environmental feedback from spatially removed locations. For example, the sector web spider *Zygiella x-notata* uses a specialized thread as both a structural bridge and signal transmitter to monitor web vibrations from its retreat at the web perimeter. To unravel this model multifunctional system, we investigated *Zygiella*'s signal thread structure with a range of techniques, including tensile testing, laser vibrometry, electron microscopy and behavioural analysis. We found that signal threads varied significantly in the number of filaments; a result of the spider adding a lifeline each time it runs along the bridge. Our mechanical property analysis suggests that while the structure varies, its normalized load does not. We propose that the signal thread represents a complex and fully integrated multifunctional structure where filaments can be added, thus increasing absolute load-bearing capacity while maintaining signal fidelity. We conclude that such structures may serve as inspiration for remote sensing design strategies.

## 1. Introduction

Substrate-borne vibrations are a common source of information for a wide range of animals and plants [1–3]. Given their universality, monitoring these vibrations is important for both communication and predator–prey interactions [1]. A model system to study the importance of vibration sensing is the spiders, as they are typically highly haptic animals, possess sensitive vibration detectors on their legs and are well known to respond to vibrations [4–6]. Web-building spiders employ their web structure as a vibration transmission platform in addition to its function as a snare [7–10]. Most spiders sit on the web itself in order to best monitor vibrations emanating from potential mates, competing conspecifics, prey or predators [11–14]. However, some spiders adopt a remote sensing technique by using a silk signal thread, which allows them to hide in a retreat safe from predators and parasitoids [12,15], with the associated cost of extra time required to sense, monitor and locate web vibrations [16].

**Figure 1.**
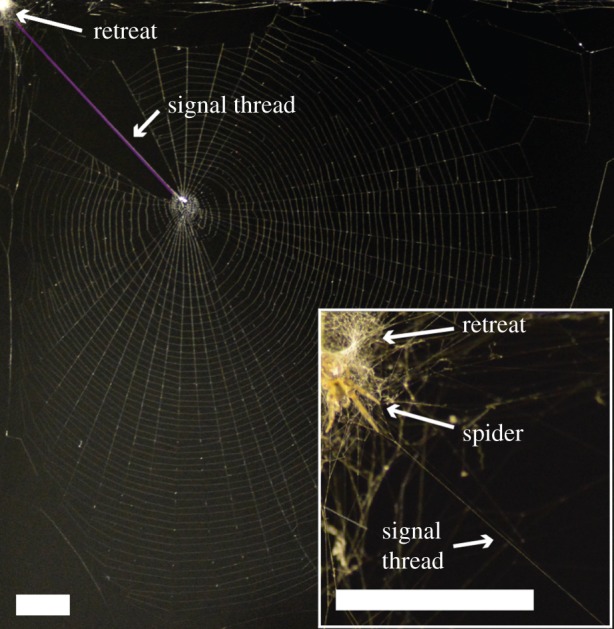
Typical web from *Zygiella x-notata*. The web is a modified orb web; a sector is kept free in a top corner, where the signal thread (purple) runs from the hub to a retreat. Inset is a spider at the retreat in resting position, with front leg on the signal thread. White bars denote 20 mm. (Online version in colour.)

*Zygiella x-notata,* the sector web spider, builds a modified orb web with a mesh-free sector and a radius thread that acts as a signalling thread (figure 1). This signal thread is built last during web construction and connects the central hub with the retreat [17], where the spider sits with one or two front legs monitoring the vibration of the signal thread (figure 1 inset) [16]. The spider will run to the hub along the signal thread when prey hits its web or in response to web vibration, indicating the signal thread's use to transmit vibrational sensory information to the spider [16,18]. As it moves along, the animal drags behind its dragline/lifeline, thus adding that to the signal thread [16] with possible implications for signal transduction and information transmission. While previous studies have examined *Zygiella* web-building behaviour [17,19–21], none have focused on the structure and properties of the signal thread itself, which is the goal of this investigation.

The signal thread is made of the same material as the radial threads, i.e. dragline silk [22,23], commonly thought to contain mostly major ampullate silk, with some evidence (occasionally) of additional minor ampullate filaments [24–26]. Multiple filaments affect the vibrational properties of silks [9], but they also affect the mechanical response of the thread. With implications for both vibrational and mechanical responses, the tension of the signal thread is higher than that of other radials [22]. Specifically, tensioning of the signal thread will directly affect transverse vibrational responses, as well as indirectly affecting longitudinal wave properties through changes in storage modulus [9]. Tension is also important for the signal thread's primary function, which is the transmission of vibrations from the hub with minimal loss of information and energy [27].

This paper outlines the structure of the signal thread by firstly examining the properties of its constituents, i.e. dragline silk bundles. A combination of tensile testing, microscopy, behavioural measurement and laser vibrometry allows us to assess the constraints on the structure of the signal thread and the implications for vibration transmission.

## 2. Methods

### 2.1. Sample preparation

Spider silks from *Z. x-notata* were either collected by forced reeling immobilized spiders [28,29] or taken from naturally spun web silks [26]. The spiders were collected in Oxford city and housed in 30 × 30 × 5 cm Perspex frames, kept in laboratory conditions (*ca* 20°C, 40% RH and a 16 L : 8 D cycle). Spiders were fed (*Drosophila*) on their web after which it was collapsed for the spider to re-ingest and re-build. Signal threads were taken from webs that followed an initial set-up phase of at least three webs [30]. Webs were photographed before removing the signal thread. Dividers fixed onto a micromanipulator were used to remove the signal thread from these established webs. In all cases, two 12.5 mm thread specimens kept under natural web tension were transferred onto cardboard frames.

After signal threads were taken from newly built webs, the spiders’ thread repair behaviour was filmed with a Panasonic HDC-TM700 HD at 50 fps. In particular, the number of times the spider moves between the hub and retreat was recorded, termed ‘number of runs'.

For forced reeling, a large *Zygiella* (0.0312 g) was selected and anaesthetized in carbon dioxide for 3 min, then caged dorsal-side-down using pins. The number of filaments and the type of silk (major and/or minor ampullate silk) were determined by observing spider spinnerets under a dissection microscope (Olympus SZ40, Tokyo, Japan), while the spider was reeled at a constant reeling speed of 20 mm s^−1^, similar to the average natural spinning speed [29]. For all spiders, photographs were taken (for size), and weight was recorded.

### 2.2. Signal thread characterization

*Zygiella* forced reeled (one spider, *n* = 10) and signal thread silks (nine spiders, *n* = 63) were mechanically deformed in a Zwick tensile tester (5 N load cell, Z0.5, Zwick GMBH, Germany) at a rate of 40% min^−1^. For specific specimens (*n* = 5 spiders, *n* = 16 threads), the pre-tension was measured by closing the clamps to find zero load. Slack on the sample was removed from the raw data to allow alignment of the load–strain curves. As the cross-sectional area is difficult to measure (although it can be estimated), load data were analysed instead of stress data.

For diameter measurements, threads were imaged under frame-tension in a scanning electron microscope (SEM; Neoscope 2000, Nikon Instruments, UK) at 10 kV high vacuum following sputter coating for 150 s at 18 mA with gold/palladium (Quorum Technologies SC7620), giving a coating of 12.5 nm. Broken ends from stress–strain experiments were also imaged to measure the approximate number of filaments (with two repeats per signal thread), examples shown in electronic supplementary material, figure S1. Average thread diameter measurement from broken ends will be an underestimate as threads will have been pulled apart.

Selected signal threads were characterized for their vibrational properties using a laser Doppler vibrometer system (PSV 300, Polytec), fitted with a close-up unit (OFV 056), as described in detail elsewhere [9]. *Zygiella* specimens were kept under tension in a Deben microtest tensile stage (2 N load cell, Deben, UK), and the first reading was taken straight after the frame was cut, under ‘natural tension’. For strain control, further tension was then added by extending the specimen using the tensile tester. Pre-load was calculated by using the post-break tension at zero load. Signal threads were measured for their transverse sonic properties in the middle of the silk thread, which was vibrated using amplified sound transmitted from a loud speaker over frequencies of 1–30 kHz. A ∼55 dB sound pressure level was used, measured using a reference microphone (Brüel & Kjaer 4138). Data from the laser vibrometer were fast-Fourier transform filtered (lowpass mode, using an ideal filter, cut-off 0.004 s) and the baseline (defined as average magnitude 25–30 kHz) was calculated. The magnitude versus frequency data were plotted by deducting the magnitude of threads at higher tension from the magnitude data of the same threads at lower tension (figure 5). Published wavespeed data on silks [9] allowed us to use the peaks at expected frequencies to estimate the fundamental mode. The peak-finding algorithm used a 40% height increase over 20 points, where adjoining peaks were interpreted as one peak. The frequency of the highest section of a fundamental mode peak was used to calculate the wavespeed.

### 2.3. Statistics

The number of runs and other parameters were correlated using Microsoft Excel, which calculates an *R*^2^-value, where 1 is a perfect linear relationship and 0 is a random relationship. Spearman's rank coefficient (*ρ*) was also calculated to quantify the correlation between the two terms, as the assumptions of the more powerful Pearson's correlation were not met. Significance, here defined as *p* < 0.05, was determined by reference to a critical values table [31].

## 3. Results and discussion

### 3.1. Forced reeled silk bundle structure and properties

**Figure 2.**
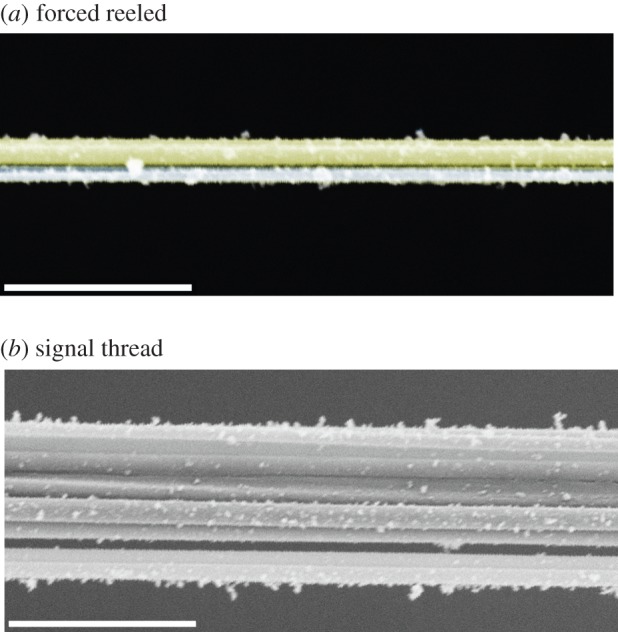
SEM image of *Zygiella* silks: (*a*) false colour SEM of forced reeled silks, where a double of larger silk (yellow; assumed major ampullate silk) and a double of smaller silk (green; assumed minor ampullate silk) are coextruded, and (*b*) naturally spun signal thread taken from a new web and kept under tension, where multiple silks are used. White bar denotes 10 µm. The fine particles are ‘dust’, presumably attracted to the silks owing to electrostatic charging [32]. (Online version in colour.)

Our SEM observations showed that signal threads always contain multiple silk filaments (figure 2). To help determine the exact number of filaments, we used forced reeled *Zygiella* silk as reference. Forced reeled silks are of a known type, the number of filaments can be controlled, and it is made from the same silks as the signal thread. As our observations (from both dissection and SEM) showed, forced reeled silk bundles are two major and two minor ampullate silk filaments (figure 2*a*).

**Figure 3.**
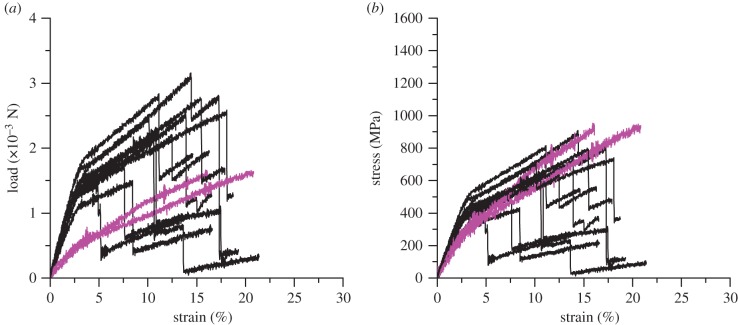
Tensile responses of 10 bundles of ampullate silks forced reeled from *Zygiella*: (*a*) load and (*b*) stress, where pink curves (where the load modulus is half) use half the total area, and black curves use the whole area. (Online version in colour.)

Forced reeled silk has a known number of fibres and thus their load–extension curves can be used to calibrate the number of filaments in load–extension curves of silk bundles of unknown composition, assuming load correlates with the number of filaments. The load–strain and stress–strain responses of forced reeled *Zygiella* silks are shown in figure 3. Some filaments showed double the load modulus (and breaking load) of others. Area allocation of the whole (two major and two minor) or half the area aligned the stress–strain curves (figure 3*b*), implying that load data (modulus and maximum load) can be used as a proxy for the number of filaments present. This variation in load was probably owing to breaking of filaments during sample mounting. There is evidence of slightly different stress–strain contours for double (black) versus single (pink) fibres after alignment (figure 3*b*), which will be due to mechanical interaction between two fibres that would increase stiffness [33]. Interestingly, the number of breaking points in figure 3 (defined by a sharp reduction in load) varied from one to three, hence the signal thread did not always break as a whole, nor did filaments break individually.

This provides important insights into the composition of *Zygiella* signal threads. In terms of mechanical response, the stress–strain contours of major ampullate silks are similar to other species [34]. However, for *Zygiella*, the minor ampullate silk contours are also similar to major ampullate silk, which is quite unlike other species [35]. For example, in *Nephila,* the minor ampullate silks have higher extensions notable during tensile testing of major/minor dragline bundles; furthermore, in this species, consistent stresses are taken by major and minor silk filaments as they break independently (electronic supplementary material, figure S2). In contrast, *Zygiella* major or minor filaments break around 15% strain, with variation in the load taken by different filaments following the initial break, indicating more variability in filament strength of both silk types. Therefore, bundling of major and minor ampullate silks leads to thread ‘ropes' composed of filaments with different diameters, thus giving varied responses to tension. Also of importance in this context is the supercontraction ability of major ampullate silk, which is lacking in minor ampullate [36,37], where the major ampullate silk will contract up to 30% when exposed to high humidity [36]. This supercontraction provides major ampullate silk with a wide range of stiffnesses, directly influencing longitudinal waves, and providing the spider with a mechanism to closely control tension through pulling supercontracted fibres, directly influencing transverse waves [9]. In combination with minor ampullate silk, which does not contract, this sets up a system which could be stable or tunable, depending on the ratio of major to minor ampullate silk filaments.

### 3.2. Comparison with signal thread structure and properties

Compared with the forced reeled control threads, the signal threads taken from *Zygiella* webs had many more silk filaments. Signal threads also had fibres of varying diameters, which supports our hypothesis that major and minor ampullate silks are bundled during signal thread construction (figure 2*b*). For naturally spun signal threads (i.e. not repair), the number of silk filaments varied from 8 to 16 filaments, as shown in the SEM images of broken ends after tensile testing (see electronic supplementary material, figure S1). Repaired signal threads showed a broader range of silk filaments present, varying from 4 to 14. Although the diameter of a single silk filament had only a weak (and non-significant) correlation with spider weight (*R*^2^ = 0.80, *ρ*_7_ = 0.70, *p* > 0.05), the *total* diameter of the signal thread did not correlate with spider size (neither weight nor carapace width, *R*^2^ = 0). These data suggest that signal thread size is not constrained by spider size and both signal thread building and repair are flexible behaviours not rigidly controlled between spiders or within an individual.

**Figure 4.**
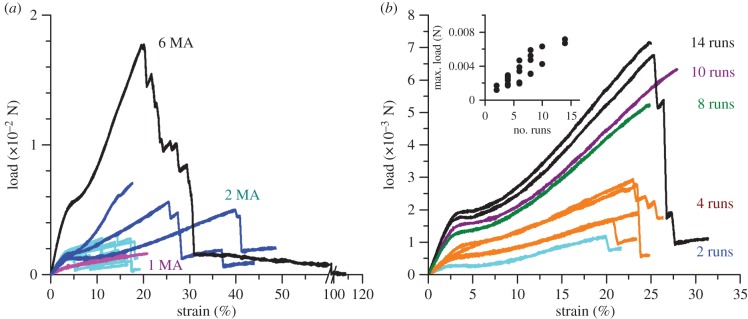
Load–strain curves of *Zygiella* silks: (*a*) forced reeled and signal threads from the same spider, where pink shows the curve where 1 MA (major ampullate silk) is taking the load, blues are 2 MA (cyan forced reeled, dark blue signal thread) and black is 6 MA (minor ampullate silk is assumed to be coextruded with MA). (*b*) Load–strain curves of signal threads from one spider, where the number of runs was recorded. Cyan is two runs, orange is four runs, green eight runs, purple 10 runs and black 14 runs, which correlate with initial modulus and maximum load (in N). Inset is a scatter graph showing maximum load versus number of runs from signal threads from multiple spiders. (Online version in colour.)

Expanding the methodology used in figure 3 to infer the number of filaments present from the tensile load data, figure 4 compares the load–extension curves of dragline silk bundles to signal threads taken from the same spider (figure 4*a*), and the effect of the number of runs on the load–extension curves taken from the same spider (figure 4*b*).

Signal threads displayed higher breaking loads and higher load moduli than their forced reeled counterpart control owing to the use of multiple filaments (figure 4*a*). Post-yield hardening was also a factor—an effect that can be caused by the interaction of strands together, whether mechanically or electrostatically [32,38]. Furthermore, the number of spider runs along the signal thread correlates closely with the load modulus (*R*^2^ = 0.70, *ρ*_24_ = 0.84, *p* < 0.01) and maximum load (inset figure 4*b*; *R*^2^ = 0.77, *ρ*_24_ = 0.87, *p* < 0.01). Therefore, as the animal moves on the signal thread, more dragline silk bundles are integrated into the signal thread. Although statistically significant, the variation seen in the relationship between number of runs and mechanical properties suggests that differing numbers of filaments may be added per run, most probably owing to the additional use of minor ampullate silk filaments.

In support of the load data, the number of runs also correlated with the number of broken ends seen in the SEM (*R*^2^ = 0.64, *ρ*_23_ = 0.87, *p* < 0.01), whereas the number of breaks seen in the stress–strain response had no relationship (*R*^2^ = 0.03, *ρ*_24_ = −0.19, *p* > 0.05). Variability in the number of breaks provides insights into functional aspects of the system. Concurrent breaking of multiple filaments points to interactions within the signal thread structure, allowing the sharing of load and cracks to propagate between filaments. Conversely, breaking in separate units shows that filaments can become separated from each other and that some filaments can take more load than others during tensile testing.

In combination, our data allow us to infer the structure of the *Zygiella* signal thread whereby multiple silk strands of bundled (i.e. co-spun) major and minor ampullate silks vary in filament number between webs, even between webs of the same individual. Further studies are needed to confirm the presence of major and minor ampullate silk in the signal thread and how they are co-spun. The signal thread structure is therefore remarkably unconstrained, with movement of the spider to and from the retreat during prey capture leading to thicker signal thread ropes as more silk filaments are recruited. This suggests that the signal thread's structure in terms of number and size of constituent filaments may well be relatively unimportant for its signalling function.

### 3.3. Implications of signal thread structure

The number and size of various filaments in a signal thread will have implications for the thread's signalling function as it affects the thread's static stress, i.e. its load normalized by its cross-sectional area. Static stress has a direct effect on transverse wave properties, it also has an indirect effect on longitudinal wave properties and it will also affect damping of vibrations [9]. The observed variability in signal thread structure, therefore, raises two questions: first, on how static stress differs between signal threads of different sizes and second, whether different filaments within an individual signal thread contribute evenly to static stress.

Regarding static stress, thread pre-tension (absolute load) correlated with the load modulus (*R*^2^ = 0.77, *ρ*_14_ = 0.65, 0.05 < *p* < 0.01), which (as we have shown) also correlated with the number of runs (figure 4). A difference also occurred between repaired and new signal threads in terms of their pre-tension, with new signal threads (the natural condition) having higher pre-tensions. It seems that the spider tensioned additional silk filaments as they were added, thus increasing the total absolute load as the signal thread increased in size.

Importantly, this lead to similar static stresses (normalized loads) between signal threads of different sizes, regardless of the size, type or number of filaments added. Such a constancy of static stress would mean that sonic properties related to time (such as propagation speed and resonant frequency) will be consistent; for example, transverse wavespeed of the signal thread will be equal to the square root of stress divided by density [9]. However, the effect of static stress on damping is a complicated matter that needs further study—interplay between filament diameter, wave type, tension and silk structure would affect vibration amplitude [27,39,40].

**Figure 5.**
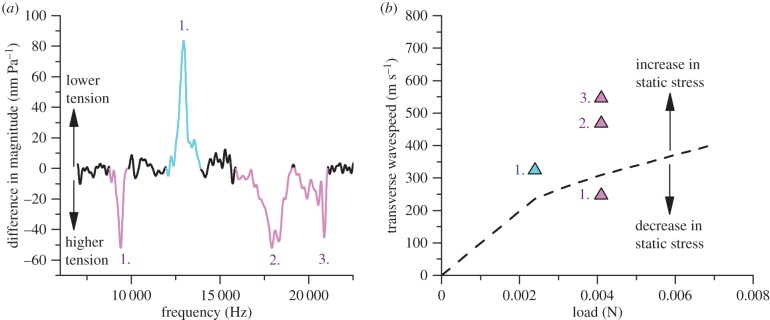
The vibrational properties of a *Zygiella* signal thread: (*a*) difference in magnitude plot, where the magnitude of a signal thread at high tension is subtracted from the same signal thread at low tension. There is one resonant peak at 0.0024 N (blue) and three at 0.0041 N (pink). Light colours give the data determined to be a peak, which are labelled with numbers. (*b*) Transverse wavespeed versus load, using the data points in panel (*a*). The dashed curve gives a theoretical relationship, where transverse wavespeed is the square root of stress divided by density. The cross-sectional area used to calculate static stress is based on an estimation from SEM measurement. Data points below this line have a lower static stress than theory, and above have a higher static stress than theory. (Online version in colour.)

Our data so far suggest multiple filaments in the signal thread integrate and interact with one another. This is supported by post-strain hardening, tensile break points (figure 3*a,b*), and additionally SEM images of signal threads (figure 2*b*). To further investigate whether individual filaments contribute evenly to static stress within the signal thread, we measured signal thread transverse resonance over a range of pre-tensions (figure 5). Using standing waves, the thread will vibrate at high amplitude at preferred frequencies (known as resonance), similar to a guitar string. At a fixed filament length, the wavespeed is proportional to the resonant frequency (transverse wavespeed is calculated in figure 5*b*). Therefore, resonance in the transverse direction (i.e. displacement perpendicular to fibre axis) provides a useful, non-destructive way to measure static stress in bundles of filaments [9]. One resonant frequency peak indicates that the signal thread is vibrating as a whole, whereas several peaks indicate that different units of the thread are vibrating independently. The change in resonant properties over tension tells us whether the signal thread structure is altering.

Thus, it appears signal threads can have multiple, separate resonant peaks not caused by harmonics (multiples of resonant frequencies), which has not been reported in previous research on spider silks [9]. This means that the multiple filaments in the signal thread are able to have different static stresses, caused either by different cross-sectional areas, and/or tension being shared by the filaments unevenly. Specifically for this signal thread, more load correlates with a simultaneous increase and decrease in static stress (figure 5*b*). This leads us to propose that some filaments were separating and vibrating independently. However, resonant peak splitting was seen at static stresses beyond yield (after the kink in the dashed curve), which is likely to have also introduced the multiple break points seen during the tensile testing. Our data on pre-load and previous work [22] suggest that the signal threads are kept at loads below the yield point under natural conditions.

Our evidence, therefore, points towards an integrated filament structure at natural tensions, where thin filaments (0.3–1.2 µm in diameter) are attracted and attached to each other (most probably electrostatically [32]) and thus share tension to create a constant static stress. What does this mean for information flow along a signal thread? Signal threads should generally vibrate as a whole, and as more filaments are added, they are integrated into the signal thread structure with little to no change in static stress. From a biological perspective, signal thread structure is beneficial for propagation times along the signal thread—there will be no change in propagation speed as more filaments are added. It also means that usually a *single* signal is received by the spider, regardless of signal thread size, which leads to simpler processing requirements for the spider's nervous system and more accurate, rapid responses to information transmitted through the web.

In terms of potential applications, the signal thread structure inspires remote sensing technologies. Regarding passive remote sensing, silks offer high fidelity signal transmission [9], and this paper has shown that the signal thread structure itself also contributes towards stable signal transmission properties. Furthermore, the structure also could be applied in active remote sensing applications, for example through implementation in piezoelectric energy harvesting materials and structures, where deformation (for example from vibration) is transformed into electricity [41]. Inspired by the signal thread design, these piezoelectric fibre bundles could come in a variety of sizes for different contexts, from microelectromechanical systems (MEMS), to large cables for civil applications [42], where deformation response can be closely controlled through bundle tensioning. Use of coated silks (e.g. zinc oxide nanowires [41]) in composite energy harvesting systems could allow tuning of fibre moduli to be implemented for different contexts [43], and silk's high toughness could additionally be useful in vibration damping applications [44].

## 4. Conclusion

A combination of tensile testing, electron microscopy and laser vibrometry has shown us that the signal thread of *Z. x-notata* is flexible in composition with apparently weak selection pressures to conserve the number of filaments present. Signal thread function would be unaffected by thread dimensions, because propagation speeds remain constant, being a function of static stress. Our data support the hypothesis that the signal thread is a fully integrated structure, which vibrates as a whole under natural static stresses. This allows the spider to readily respond to signal thread vibration, regardless of its size, with no cost of time delay or extra signal processing required. This suggests that the signal thread represents a complex, multifunctional, sensory structure allowing for variation in structural or load-bearing performance, while maintaining signal fidelity. From an industrial perspective, it might serve as inspiration for future development of multifunctional remote sensing materials and structures.

## Supplementary Material

Figure S1.

## Supplementary Material

Figure S2.

## Data accessibility

Some data supporting this article have been uploaded as part of the electronic supplementary material. Any further data can be requested from the corresponding author.

## References

[1] Hill PSM 2009 How do animals use substrate-borne vibrations as an information source? Naturwissenschaften 96, 1355–1371. (10.1007/s00114-009-0588-8) 19593539

[2] Appel HM, Cocroft RB 2014 Plants respond to leaf vibrations caused by insect herbivore chewing. Oecologia 175, 1257–1266. (10.1007/s00442-014-2995-6) 24985883 PMC4102826

[3] Gagliano M, Mancuso S, Robert D 2012 Towards understanding plant bioacoustics. Trends Plant Sci. 17, 323–325. (10.1016/j.tplants.2012.03.002) 22445066

[4] Hebets EA, Elias DO, Mason AC, Miller GL, Stratton GE 2008 Substrate-dependent signalling success in the wolf spider, *Schizocosa retrorsa*. Anim. Behav. 75, 605–615. (10.1016/j.anbehav.2007.06.021)

[5] Barth FG, Geethabali. 1982 Spider vibration receptors: threshold curves of individual slits in the metatarsal lyriform organ. J. Comp. Physiol. 148, 175–185. (10.1007/BF00619124)

[6] Szlep R 1964 Change in the response of spiders to repeated web vibrations. Behaviour 23, 203–239. (10.1163/156853964X00157)

[7] Lin LH, Edmonds DT, Vollrath F 1995 Structural engineering of an orb-spider's web. Nature 373, 146–148. (10.1038/373146a0)

[8] Masters WM, Markl H 1981 Vibration signal transmission in spider orb webs. Science 213, 363–365. (10.1126/science.213.4505.363) 17819912

[9] Mortimer B, Gordon SD, Siviour CR, Holland C, Vollrath F, Windmill JFC 2014 The speed of sound in silk: linking material performance to biological function. Adv. Mater. 26, 5179–5183. (10.1002/adma.201401027) 24902950 PMC4140601

[10] Blackledge TA, Kuntner M, Agnarsson I 2011 The form and function of spider orb webs: evolution from silk to ecosystems. In Advances in insect physiology, vol. 41 (ed Casas J), pp. 175–262. London, UK: Academic Press.

[11] Tarsitano M, Jackson RR, Kirchner WH 2000 Signals and signal choices made by the araneophagic jumping spider *Portia fimbriata* while hunting the orb-weaving web spiders *Zygiella x-notata* and *Zosis geniculatus*. Ethology 106, 595–615. (10.1046/j.1439-0310.2000.00570.x)

[12] Foelix RF 2010 Biology of spiders, p. 330, 3rd edn Oxford, NY: Oxford University Press.

[13] Maklakov AA, Bilde T, Lubin Y 2003 Vibratory courtship in a web-building spider: signalling quality or stimulating the female? Anim. Behav. 66, 623–630. (10.1006/anbe.2003.2245)

[14] Soley FG, Taylor PW 2013 Ploys and counterploys of assassin bugs and their dangerous spider prey. Behaviour 150, 397–425. (10.1163/1568539x-00003059)

[15] Pasquet A, Cardot J, Leborgne R 2007 Wasp attacks and spider defence in the orb weaving species *Zygiella x-notata*. J. Insect Behav. 20, 553–564. (10.1007/s10905-007-9098-8)

[16] Klärner D, Barth FG 1982 Vibratory signals and prey capture in orb-weaving spiders (*Zygiella x-notata, Nephila clavipes*, Araneidae). J. Comp. Physiol. 148, 445–455. (10.1007/BF00619783)

[17] Zschokke S, Vollrath F 1995 Web construction patterns in a range of orb-weaving spiders (Araneae). E. J. Entomol. 92, 523–541.

[18] Liesenfeld FJ 1956 Untersuchungen am netz und über den erschütterungssinn von *Zygiella x-notata* (CL) (Araneidae). Z. Vergl. Physiol. 38, 563–592. (10.1007/bf00341110)

[19] Pasquet A, Ridwan A, Leborgne R 1994 Presence of potential prey affects web-building in an orb-weaving spider *Zygiella-x-notata*. Anim. Behav. 47, 477–480. (10.1006/anbe.1994.1066)

[20] Venner S, Pasquet A, Leborgne R 2000 Web-building behaviour in the orb-weaving spider *Zygiella x-notata*: influence of experience. Anim. Behav. 59, 603–611. (10.1006/anbe.1999.1327) 10715183

[21] Venner S, Bel-Venner MC, Pasquet A, Leborgne R 2003 Body-mass-dependent cost of web-building behavior in an orb weaving spider, *Zygiella x-notata*. Naturwissenschaften 90, 269–272. (10.1007/s00114-003-0420-9) 12835838

[22] Wirth E, Barth FG 1992 Forces in the spider orb web. J. Comp. Physiol. A 171, 359–371. (10.1007/BF00223966)

[23] Denny M 1976 The physical properties of spider's silk and their role in design of orb-webs. J. Exp. Biol. 65, 483–506.

[24] Peters HM 1990 On the structure and glandular origin of bridging lines used by spiders for moving to distant places. Acta Zool. Fenn. 190, 309–314.

[25] Peters HM 1993 Functional organization of the spinning apparatus of *Cyrtophora citricola* with regard to the evolution of the web (Araneae, Araneidae). Zoomorphology 113, 153–163. (10.1007/BF00394856)

[26] Hesselberg T, Vollrath F 2012 The mechanical properties of the non-sticky spiral in *Nephila* orb webs (Araneae, Nephilidae). J. Exp. Biol. 215, 3362–3369. (10.1242/jeb.068890) 22735349

[27] Main IG 1993 Vibrations and waves in physics, 3rd edn Cambridge, UK: Cambridge University Press.

[28] Madsen B, Vollrath F 2000 Mechanics and morphology of silk drawn from anesthetized spiders. Naturwissenschaften 87, 148–153. (10.1007/s001140050694) 10798202

[29] Vollrath F, Madsen B, Shao ZZ 2001 The effect of spinning conditions on the mechanics of a spider's dragline silk. Proc. R. Soc. Lond. B 268, 2339–2346. (10.1098/rspb.2001.1590) PMC1088885 11703874

[30] Zschokke S, Herberstein ME 2005 Laboratory methods for the maintaining and studying web-building spiders. J. Arachnol. 33, 205–213. (10.1636/CT04-72.1)

[31] Buqué G 2001 Statistics. Oxford, UK: Heinemann.

[32] Vollrath F, Edmonds D 2013 Consequences of electrical conductivity in an orb spider's capture web. Naturwissenschaften 100, 1163–1169. (10.1007/s00114-013-1120-8) 24323174

[33] Cranford SW 2013 Increasing silk fibre strength through heterogeneity of bundled fibrils. J. R. Soc. Interface 10, 1–13. (10.1098/rsif.2013.0148) PMC3627094 23486175

[34] Swanson BO, Blackledge TA, Beltran J, Hayashi CY 2006 Variation in the material properties of spider dragline silk across species. Appl. Phys. A, Mater. Sci. Process. 82, 213–218. (10.1007/s00339-005-3427-6)

[35] Guinea GV et al. 2012 Minor ampullate silks from *Nephila* and *Argiope* spiders: tensile properties and microstructural characterization. Biomacromolecules 13, 2087–2098. (10.1021/bm3004644) 22668322

[36] Liu Y, Sponner A, Porter D, Vollrath F 2008 Proline and processing of spider silks. Biomacromolecules 9, 116–121. (10.1021/bm700877g) 18052126

[37] Gatesy J, Hayashi C, Motriuk D, Woods J, Lewis R 2001 Extreme diversity, conservation, and convergence of spider silk fibroin sequences. Science 291, 2603–2605. (10.1126/science.1057561) 11283372

[38] Madsen B, Hoffmeyer P, Thomsen AB, Lilholt H 2007 Hemp yarn reinforced composites—I. Yarn characteristics. Compos. A, Appl. Sci. Manuf. 38, 2194–2203. (10.1016/j.compositesa.2007.06.001)

[39] Frohlich C, Buskirk RE 1982 Transmission and attenuation of vibration in orb spider webs. J. Theor. Biol. 95, 13–36. (10.1016/0022-5193(82)90284-3)

[40] Landolfa MA, Barth FG 1996 Vibrations in the orb web of the spider *Nephila clavipes*: cues for discrimination and orientation. J. Comp. Physiol. A 179, 493–508. (10.1007/BF00192316)

[41] Masghouni N, Burton J, Philen MK, Al-Haik M 2015 Investigating the energy harvesting capabilities of a hybrid ZnO nanowires/carbon fiber polymer composite beam. Nanotechnology 26, 095401 (10.1088/0957-4484/26/9/095401) 25670370

[42] Silleto MN, Yoon SJ, Arakawa K 2015 Piezoelectric cable macro-fiber composites for use in energy harvesting. Int. J. Energy Res. 39, 120–127. (10.1002/er.3227)

[43] Mortimer B, Guan J, Holland C, Porter D, Vollrath F 2015 Linking naturally and unnaturally spun silks through the forced reeling of *Bombyx mori*. Acta Biomater. 11, 247–255. (10.1016/j.actbio.2014.09.021) 25242653

[44] Choudhary N, Kaur D 2015 Vibration damping materials and their applications in nano/micro-electro-mechanical systems: a review. J. Nanosci. Nanotechnol. 15, 1907–1924. (10.1166/jnn.2015.10324) 26413606

