# Supplementary figures and images for: Unpicking the signal thread of the sector web spider Zygiella x-notata
Source: J R Soc Interface. 2015 Dec 6;12(113):20150633. doi: 10.1098/rsif.2015.0633 (PMC4707845; doi:10.1098/rsif.2015.0633)

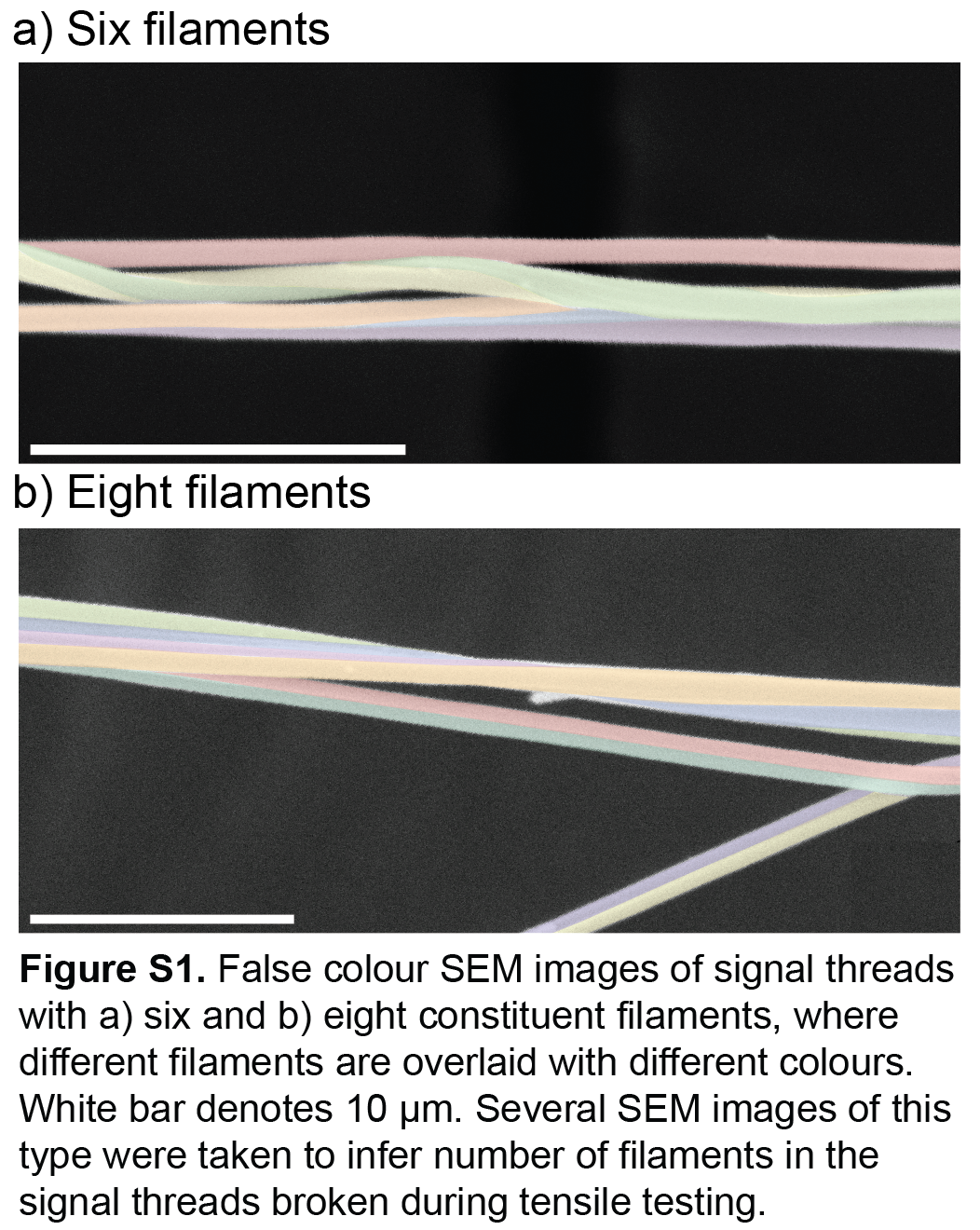

Supplement: Figure S1. [file rsif20150633supp1.tif]

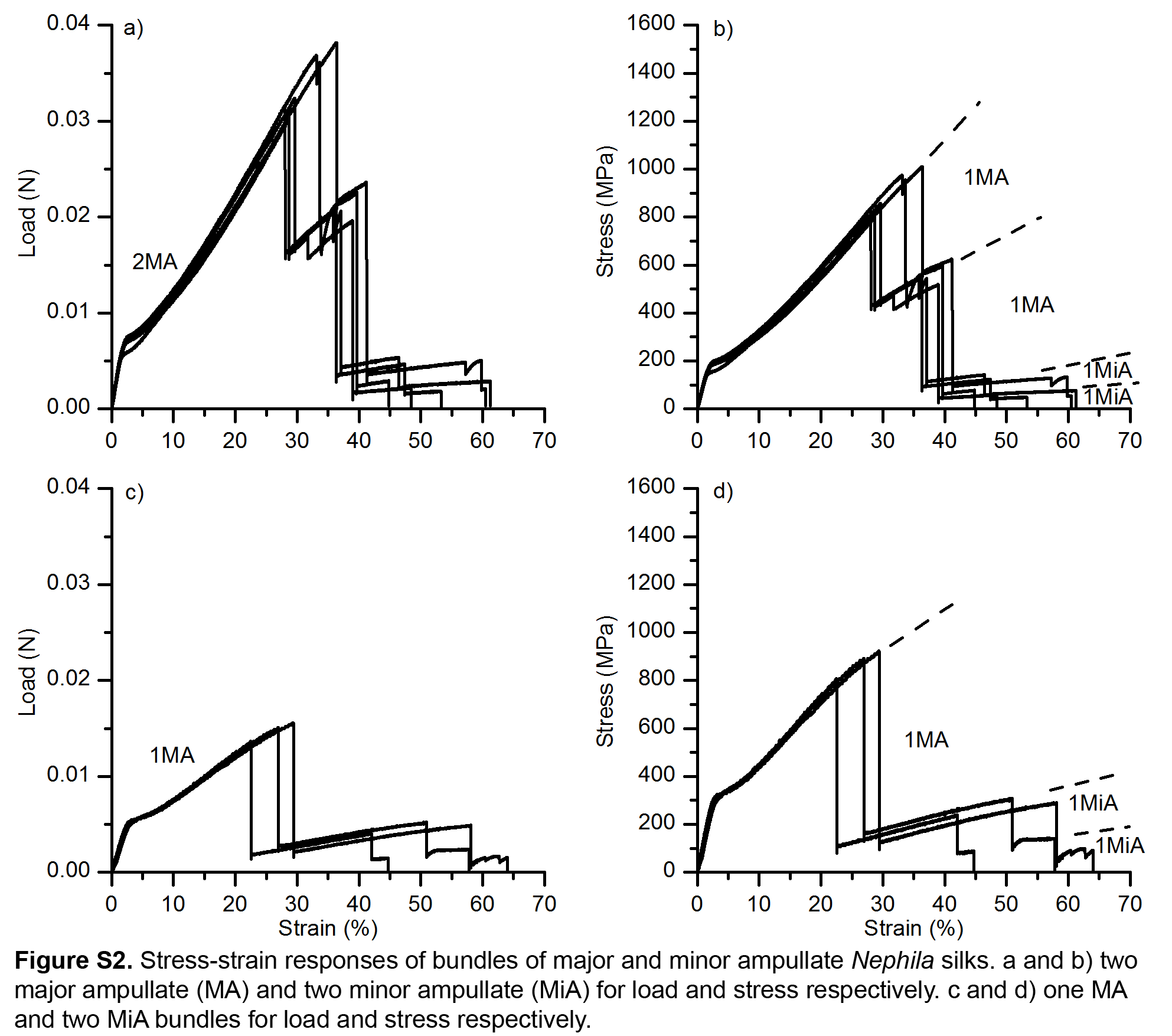

Supplement: Figure S2. [file rsif20150633supp2.tif]
